# Neuroprotective and Antioxidant Enhancing Properties of Selective *Equisetum* Extracts

**DOI:** 10.3390/molecules26092565

**Published:** 2021-04-28

**Authors:** Denisa Batir-Marin, Monica Boev, Oana Cioanca, Cornelia Mircea, Ana Flavia Burlec, Galba Jean Beppe, Adrian Spac, Andreia Corciova, Lucian Hritcu, Monica Hancianu

**Affiliations:** 1 Department of Pharmaceutical Sciences, Faculty of Medicine and Pharmacy, Dunarea de Jos University, 800010 Galati, Romania; denisa.batir@ugal.ro (D.B.-M.); monica.boev@ugal.ro (M.B.); 2 Faculty of Pharmacy, Grigore T. Popa University of Medicine and Pharmacy Iasi, 700115 Iasi, Romania; cornelia.mircea@umfiasi.ro (C.M.); ana-flavia.l.burlec@umfiasi.ro (A.F.B.); adrian.spac@umfiasi.ro (A.S.); maria.corciova@umfiasi.ro (A.C.); monica.hancianu@umfiasi.ro (M.H.); 3 Department of Biological Sciences, Faculty of Science, University of Maroua, Maroua P.O. Box 814, Cameroon; jean.beppe-galba@fs.univ-maroua.cm; 4 Department of Biology, Faculty of Biology, Alexandru Ioan Cuza University of Iasi, 700506 Iasi, Romania; hritcu@uaic.ro

**Keywords:** *Equisetum pratense*, *Equisetum telmateia*, *Equisetum sylvaticum*, UHPLC, antioxidant activity, neuroprotective, polyphenolic derivatives

## Abstract

The sterile stems belonging to the *Equisetum* species are often used in traditional medicine of various nations, including Romanians. They are highly efficient in treating urinary tract infections, cardiovascular diseases, respiratory tract infections, and medical skin conditions due to their content of polyphenolic derivatives that have been isolated. In this regard, this study aimed to provide the chemical composition of the extracts obtained from the *Equisetum* species (*E. pratense*, *E. sylvaticum*, *E. telmateia*) and to investigate the biological action in vitro and in vivo. For the chemical characterization of the analyzed *Equisetum* species extracts, studies were performed by using ultra-high-performance liquid chromatography (UHPLC-DAD). In vitro evaluation of the antioxidant activity of the plant extracts obtained from these species of *Equisetum* genus was determined. The neuroprotective activity of these three ethanolic extracts from the *Equisetum* species using zebrafish tests was determined in vivo. All obtained results were statistically significant. The results indicate that *E. sylvaticum* extract has a significant antioxidant activity; whereas, *E. pratense* extract had anxiolytic and antidepressant effects significantly higher than the other two extracts used. All these determinations indicate promising results for the antioxidant in vitro tests and neuroprotective activity of in vivo tests, particularly mediated by their active principles.

## 1. Introduction

The Horsetails (Equisetaceae) are one of the oldest groups of vascular plants. Despite the small size of the family and a limited number of morphological characters, most species of horsetails are characterized by very high morphological variability [1]. *Equisetum* L. genus is comprised of approximately 15 extant species with a nearly worldwide distribution [2,3]. Research in the past decades has shown an increased interest in phytochemical products and plant extracts, due to frequent use in the prevention and treatment of some diseases. Several studies have demonstrated that the antioxidants found in plants are of major interest to medicine since they protect the organism against oxidative stress, generated in the context of some diseases: atherosclerosis, ischemic cardiac disease, cancer, Alzheimer’s disease, Parkinson’s disease, aging, and even in infectious diseases [4,5,6]. Nevertheless, various tests are still underway for the majority of the positive results obtained in vitro and/or in vivo, and only a few are transferable to clinical uses. Furthermore, other lab data confirmed the traditional uses of medicinal and aromatic plants.

*Equisetum* species are perennial ferns from the Equisetaceae family. They have a fertile stem bearing yellowish spores, produced in early spring. The green, photosynthetic, strongly branched, sterile stems are produced in late spring and persist until late autumn. The sterile strain is the medicinal product of the *Equisetum arvense* L. plant (*Equiseti* herba) mentioned in the European Pharmacopoeia. Its well-established use is as a diuretic and is solely based on long term usage in traditional medicine. However, the present study is based on the phytochemical analysis of the chemical composition, but also on the in vitro and in vivo biological action of three different species of *Equisetum: E. pratense* Ehrh., *E. sylvaticum* L. and *E. telmateia* Ehrh. which are found in the wild flora of Romania.

The most widely known phytochemical compounds of the *Equisetum* genus are flavonoids, phenolic acids, alkaloids, phytosterols, tannins, and triterpenoids [7]. However, such compounds are common to the majority of the superior plants. Experimental data published so far and in this paper show that polyphenolic derivatives that have been isolated from *Equisetum* species can provide indirect protection by activating antioxidant transcription in the promoter regions of genes that induce oxidative stress [5,8]. Besides, polyphenols can modulate both the cellular signal involved in cell proliferation and cell development itself by inhibiting inflammation and initiating the process of apoptosis of damaged cells [9,10].

Oxygen is the essential element for maintaining cellular activities, and also for the body in general [11]. Penetrated in the form of gas, through the respiratory tract, molecular oxygen is mainly transformed into water in the biochemical processes in the respiratory chain and thus allows the production of the energy required for cellular processes, in the form of ATP [11]. Under physiological conditions, some of this oxygen is transformed into reactive oxygen species (ROS), for which neutralization systems in the form of antioxidant substances or enzymes are developed at the cellular level. Long-term or repeated exposure of the body to oxidative stress causes the development of pathological phenomena, including cancer [12,13,14]. In these situations, it is necessary to supplement the antioxidant defense capacity at the cellular level by administering antioxidant vitamins (vitamins C and E) or plant extracts that contain antioxidants [15,16]. Numerous tests to evaluate the antioxidant action in vitro have been developed over time, as a preliminary step to testing the antioxidant effects in vivo [17]. From these tests, those selected were based on antioxidant mechanisms that can also occur in the biological environment, such as chelation of ferrous ion, lipoxygenase inhibition, the scavenger capacity of the hydroxyl radical, and the superoxide radical anion [18].

Neurodegenerative diseases have become increasingly intensively studied, and their early detection is the point of interest in understanding their etiology. Most inducing processes of neurodegeneration cause the initiation of a chemical cascade that results in apoptosis or necrosis of neurons. This manifests itself in the form of loss of certain neurological and cognitive functions [19,20]. Most often, these degenerative manifestations are accompanied by regenerative or neuroprotective compensatory mechanisms such as the presence of antioxidant enzymes, neurotrophic growth factors, or peptides with a regulatory role [19]. By discovering the most common processes of neurodegeneration—and also neuroprotection—intervention strategies can be drawn in the progress of various neurodegenerative diseases [21]. Neuroprotection is a sum of strategies to protect the nervous system against neuronal damage or degeneration caused by events such as neurodegenerative diseases, cerebral ischemia, or various traumas that may occur. The goal of neuroprotection is to limit the spread of neural apoptosis and minimize neural dysfunction through mechanisms to maintain the integrity of cellular interactions [19,20,21].

The present study aims to highlight the chemical composition of the extracts obtained from the *Equisetum* species (*E. pratense*, *E. sylvaticum*, *E. telmateia*) and to investigate the biological action in vitro and in vivo.

## 2. Results and Discussion

### 2.1. Obtaining Extracts

Sterile stems of *E. pratense* Ehrh., *E. telmateia* Ehrh. and *E. sylvaticum* L. were used. Two types of total extracts were obtained using different solvents: 70% methanolic and 70% ethanolic. To obtain extracts rich in polyphenolic compounds, the extraction with these solvents was performed, taking into account the polar character of these compounds and the high extractability in such solvents.

### 2.2. Semi-Quantitative and Qualitative Determination of the Polyphenolic Compounds (UHPLC-DAD)

The presence of the following polyphenolic derivatives in the two types of extracts (methanolic and ethanolic) obtained from the three investigated *Equisetum* samples was confirmed by the liquid chromatography technique: chlorogenic acid, caffeic acid, ferulic acid, as well as glycosylated derivatives of quercetin and luteolin. Such active principles have also been identified by other authors in extracts obtained by various methods from *E. arvense* [13,17,22,23]. According to the spectral and quantitative analysis, the flavonoid components quantified in the samples are presented briefly in Table 1.

Quantitatively, for the methanolic extracts, *E. telmateia* and *E. sylvaticum* are richer in the flavonoid fraction than *E. pratense*, being evident that the glycosylated derivatives of quercetin, luteolin, and apigenin are predominant in all three samples. It is noted the low solubility of the flavonoid aglycones in the hydroalcoholic solution, but similar to the studies of other researchers, the extraction yield also varies depending on the chemical structure of each component. Quercetin glycosides are predominant in the *E. sylvaticum* sample, and in the *E. telmateia* sample, quercetin and apigenin glycosides represent the majority. The noted differences between the methanolic and ethanolic extracts for the same species are related to each compound affinity towards the solvent. Generally, higher extraction yields are obtained for phenolic acids and glycosylated flavonoids in hydroalcoholic solutions rather than organic solvents, which is observed in our results. Although the solvent is one of the main factors influencing the extraction of certain compounds, the temperature and the use of shaking or stirring can also impact the extractability. Interestingly, ultrasounds favored the extraction of quercetin-3-glucoside in *E. sylvaticum* ethanolic extract, whereas reflux extraction in methanol 70% increased the extractability of the majority of the flavonoid glycosides for all species. This can be explained to some extent taking into account that ultrasounds break molecular structures, leading probably to artefacts or more lipophilic structures which were not extracted nor identified in the investigated samples.

The concentrations of the four identified polyphenolic acids are shown in Table 2.

For the methanolic extracts, the polyphenolic acid with the highest content is chlorogenic acid, regardless of the investigated sample, but the maximum content is found in *E. telmateia* (approx. 10 mg/g), while the proportion decreases in half in *E. sylvaticum* and less than 7% in *E. pratense*. In general, the amount of polyphenol carboxylic acids is higher in hydroalcoholic extracts than in methanolic extracts, a fact proved by the high content of ferulic acid (*E. sylvaticum*) and chlorogenic acid (*E. telmateia*). Such differences could also be related to the growing environment and the biosynthetic capacity of each species. Our specimens were collected from three different regions in which the species grow usually in larger amounts. *E. sylvaticum* was collected from the highest altitude (915 m) within the spruce forest, *E. pratense* from 681 m, placed along the riverbank’s bushes and *E. telmateia* from the edges of the forests at a lower altitude (259 m). More research is still undergoing in this aspect.

### 2.3. In Vitro Evaluation of the Antioxidant Activity of the Plant Extracts Obtained from the Three Species of the Equisetum Genus

#### 2.3.1. The Chelating Capacity of the Ferrous Ion Determination

**Figure 1:**
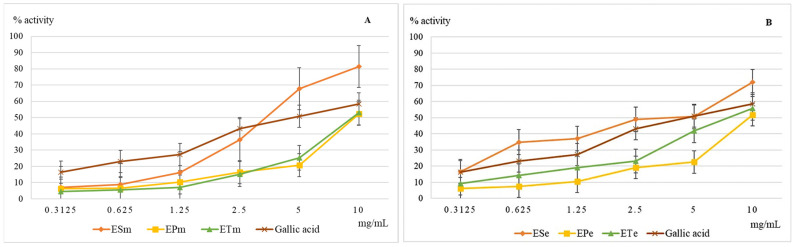
Graphical representation of the mean values (*n* = 5) of the iron-chelating capacity (%) depending on the concentration of the methanolic (**A**) and ethanolic (**B**) extracts used. Legend: ESm—*E. sylvaticum* methanolic, EPm—*E. pratense* methanolic, ETm—*E. telmateia* methanolic; ESe—*E. sylvaticum* ethanolic, EPe—*E. pratense* ethanolic, ETe—*E. telmateia* ethanolic.

The ferrous ion is present in serum and intracellular fluid in extremely small amounts, but when its concentration increases and its protein binding capacity is low, it may be involved in pathological phenomena [22]. At the cellular level, it participates in the Fenton and Haber-Weiss reactions of hydroxyl radical generation, one of the most aggressive free radicals involved in the occurrence of oxidative stress [23]. The chelating capacity of ferrous ions is an important indicator for the evaluation of the antioxidant activity. The results obtained in this test are shown in Figure 1.

At concentrations above 3 mg/mL, the methanolic extract 70% of *E. sylvaticum* is more active compared to the gallic acid, this time the phyto-complex present in the extract achieving an additional synergy compared to a single compound. The analysis of ethanolic extracts 70% indicates again the superiority of the extract obtained from *E. sylvaticum*, but, unlike the methanol one, it is not the richest in polyphenols of the three extracts analyzed. This time the difference in efficacy between the extracts of *E. telmateia* and *E. pratense* increases in favor of the first extract.

#### 2.3.2. Determination of the Lipoxygenase Inhibition Capacity

**Figure 2:**
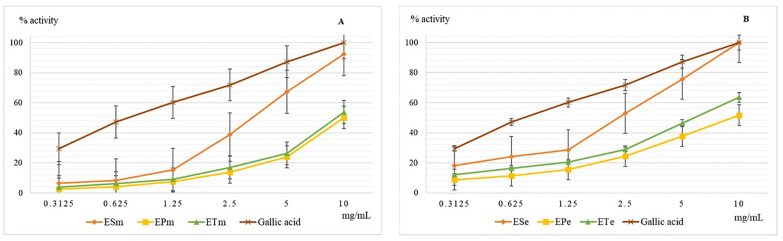
Graphical representation of the mean values (*n* = 5) of the lipoxygenase inhibition capacity (%) depending on the concentration of the methanolic (**A**) and ethanolic extracts (**B**) used. Legend: ESm—*E. sylvaticum* methanolic, EPm—*E. pratense* methanolic, ETm—*E. telmateia* methanolic; ESe—*E. sylvaticum* ethanolic, EPe—*E. pratense* ethanolic, ETe—*E. telmateia* ethanolic.

Lipoxygenase (EC.1.13.11.33) is an enzyme of the oxidoreductase class that catalyzes the oxidation of unsaturated fatty acids with the formation of peroxides [24]. They are involved in oxidative phenomena at the cellular level increasing the oxidation rate of lipids with the appearance of pathological phenomena. The enzyme is also involved in the synthesis of inflammation mediators [25]. The results obtained for the evaluation of the lipoxygenase inhibition capacity are shown in Figure 2.

EPe, the ethanolic extract from *E. pratense*, has a better inhibitory capacity against lipoxygenase than its own methanolic sample (EPm). Generally, comparing the values obtained for this test for both types of extracts, we observe that ethanolic extracts are more active than the methanolic, but lower than the chosen standard. However, at 10 mg/mL ESe proves a similar potential with gallic acid. For both methanol and ethanolic extracts, the best antioxidant activity determined for this test was obtained for the *E. sylvaticum* species, the values recorded especially for the ethanol extract were slightly higher, but comparable to those of gallic acid, used as a reference. All extracts obtained from the three species of the genus *Equisetum* showed lower efficiency than gallic acid.

#### 2.3.3. Determination of the Scavenger Action of the Hydroxyl Radical

**Figure 3:**
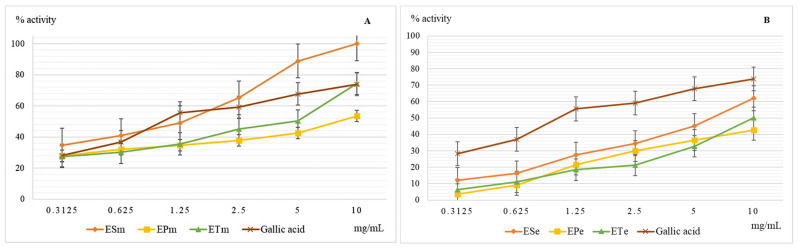
Graphical representation of the mean values (*n* = 5) of the scavenger capacity of the hydroxyl radical (%) depending on the concentration of the methanolic (**A**) and ethanolic extracts (**B**) used. Legend: ESm—*E. sylvaticum* methanolic, EPm—*E. pratense* methanolic, ETm—*E. telmateia* methanolic; ESe—*E. sylvaticum* ethanolic, EPe—*E. pratense* ethanolic, ETe—*E. telmateia* ethanolic.

The hydroxyl radical causes the initiation of oxidation reactions of unsaturated fatty acids that are present in the structure of membrane phospholipids, and their oxidation under conditions of oxidative stress will affect the stability of the cell membrane with the risk of uncontrolled loss of cell contents or reduced cell viability. Last but not least, this hydroxyl radical induces the oxidation of proteins with the modification of their spatial structure and affects their biological functions. In vitro and in vivo studies have shown the negative effect of plasma hydroxyl radical on the stability of fibrinogen which will be more easily converted to fibrin, which ultimately leads to increased blood coagulation [10,15]. The results obtained when evaluating the scavenger capacity of the hydroxyl radical are presented in Figure 3.

Methanolic extracts have a higher scavenger capacity of the hydroxyl radical compared to ethanolic ones, but without exceeding the efficiency of gallic acid used as a reference substance. The lowest scavenger activity of the hydroxyl radical was recorded for hydro-alcoholic extracts of *E. pratense*. The methanolic extract of the species *E. sylvaticum* showed the most important action, being slightly higher than that of the reference substance, gallic acid. Neutralization of the hydroxyl radical depends very much on the presence in the structure of the compound or scavenger compounds of groups capable of yielding hydrogen atoms, such as OH groups in the structure of polyphenols. In ethanolic extracts, 70%, glycosylated forms of polyphenols predominate, compared to methanolic extracts in which aglycones containing several free OH groups and capable of reducing action predominates.

#### 2.3.4. Determination of the Scavenger Capacity of the Superoxide Anion

**Figure 4:**
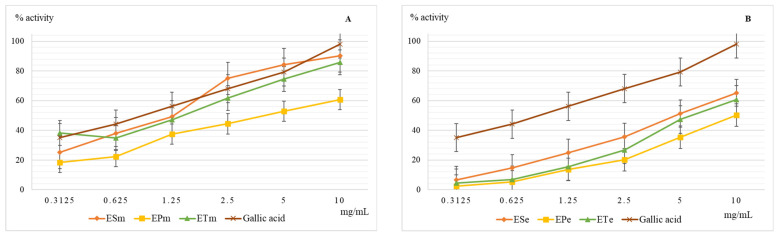
Graphical representation of the mean values (*n* = 5) of the scavenger capacity of the superoxide radical anion (%) depending on the concentration of the methanolic (**A**) and ethanolic (**B**) extracts. Legend: ESm—*E. sylvaticum* methanolic, EPm—*E. pratense* methanolic, ETm—*E. telmateia* methanolic; ESe—*E. sylvaticum* ethanolic, EPe—*E. pratense* ethanolic, ETe—*E. telmateia* ethanolic.

The superoxide radical anion is formed by the reduction of molecular oxygen that accepts a single electron, most often in the side reactions of the mitochondrial respiratory chain. Chemically, it has a medium oxidizing character but can generate hydroxyl radical and singlet oxygen which are very strong oxidants [26,27]. The results obtained when evaluating the scavenger capacity of the superoxide radical anion are presented in Figure 4.

It is noted that the antioxidant efficacy for this test is closer in value to the methanolic extracts of *E. sylvaticum* and *E. telmateia* compared to the reference substance, gallic acid. As with all other determinations of antioxidant action, *E. pratense* showed the lowest action for both methanolic and ethanolic extract. If the presence of hydrogen donor groups in the scavenger molecule is necessary to neutralize the hydroxyl radical, then both hydrogen donor groups and functional groups capable of neutralizing the anion charge are needed to neutralize the superoxide radical anion.

### 2.4. In Vivo Evaluation of the Neuroprotective Activity of the Plant Extracts Obtained from the Three Species of Equisetum

Taking into account all results, the common use of the medicinal species as tinctures, the potential use without major risks (usually given by residual solvents, such as methanol), the environment protection and, above all, the solubility of the extracts in water, we decided to evaluate the neuroprotective action only for the ethanolic extracts of *Equisetum* species (*E. pratense* Ehrh., *E. sylvaticum* L., and *E. telmateia* Ehrh.) by administering the samples to zebrafish. Moreover, in the preliminary studies we observed no toxicity or significant behavior changes in two control groups placed in a fish tank, dosed as high as 5 mg/L. The ethanolic extracts were completely soluble in the water without any residues, which is essential in testing in a water tank. In this regard, using specific behavioral tests, the effects on spatial memory (Y-maze test) and anxiety-like behavior (novel tank diving test—NTT) were followed.

#### 2.4.1. Novel Tank Diving Test (NTT)

The NTT test is used to assess the anxiety-like response. The position of the animal in the tank (at the top or bottom of the tank) is considered to be an index of anxiety [28]. For each type of the *Equisetum* ethanolic extract (EPe, ESe, ETe), individual tests were performed at both 0.5 mg/L and 1 mg/L dose.

**Figure 5:**
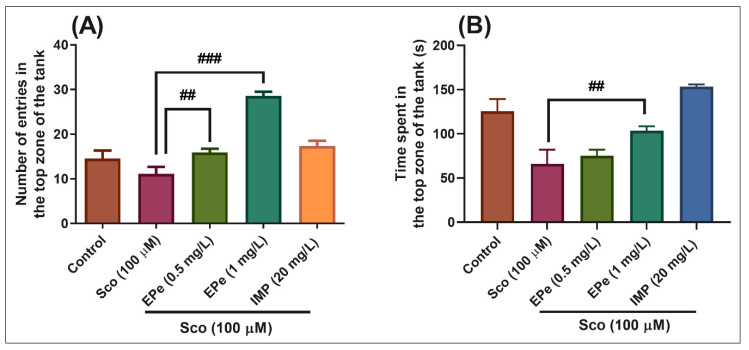
The *Equisetum pratense* ethanolic extract (EPe, 0.5 and 1 mg/L) reduced the anxiety-like response in the scopolamine (Sco, 100 μM)-induced zebrafish in the novel tank diving (NTT) test. (**A**) represent the number of entries in the top zone of the tank in different groups; (**B**) represent the time spent in the top zone of the tank (s) in different groups. For Tukey’s posthoc analysis: Sco vs. EPe (0.5 mg/L): ## *p* < 0.001; Sco vs. EPe (1 mg/L): ### *p* < 0.0001 (**A**); Sco vs. EPe (1 mg/L): ## *p* < 0.001 (**B**).

In the NTT test, one-way ANOVA demonstrated a significant effect of the treatment on the number of entries in the top zone of the tank in different groups (F(4,45) = 25.22, *p* < 0.0001) (Figure 5A), and the time spent in the top zone of the tank (F(4,45) = 12.16, *p* < 0.0001) (Figure 5B). Zebrafish exposed to scopolamine (Sco) exhibited a significant decrease in the time spent in the top zone of the tank as compared to the control group (Figure 5B, *p* < 0.001), suggesting high levels of anxiety. Moreover, treatment with EPe prevented the amnesic effect of Sco, as evidenced by increases in the number of entries in the top zone of the tank (Figure 5A, *p* < 0.001 for 0.5 mg/L and *p* < 0.0001 for 1 mg/L) as compared to Sco-alone treated group, whereas a significant increase in the time spent in the top zone of the tank was noticed for the 1 mg/L (Figure 5B, *p* < 0.001). Imipramine (IMP, 20 mg/L), a tricyclic antidepressant, was used as a reference drug in the NTT test.

**Figure 6:**
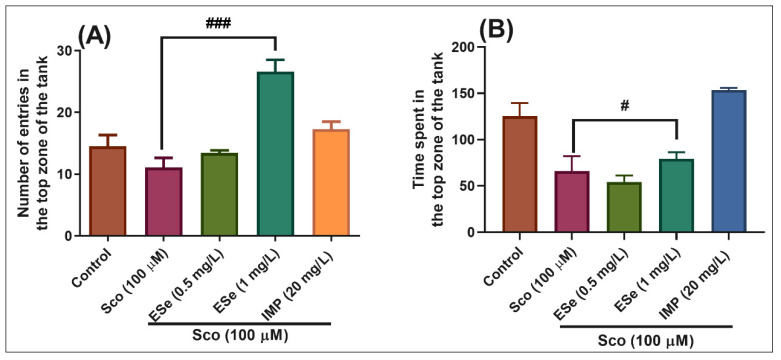
The *Equisetum sylvaticum* ethanolic extract (ESe, 0.5 and 1 mg/L) reduced the anxiety-like response in the scopolamine (Sco, 100 μM)-induced zebrafish in the novel tank diving (NTT) test. (**A**) represent the number of entries in the top zone of the tank in different groups; (**B**) represent the time spent in the top zone of the tank (s) in different groups. For Tukey’s posthoc analysis: Sco vs. ESe (1 mg/L): ### *p* < 0.0001 (**A**); Sco vs. ESe (1 mg/L): # *p* < 0.01 (**B**).

In the NTT test, one-way ANOVA demonstrated a significant effect of the treatment on the number of entries in the top zone of the tank in different groups (F(4,45) = 16.26, *p* < 0.0001) (Figure 6A), and the time spent in the top zone of the tank (F(4,45) = 15.86, *p* < 0.0001) (Figure 6B). Administration of Sco induced a significant decrease in the time spent in the top zone of the tank as compared to the control group (Figure 6B, *p* < 0.001), suggesting high levels of anxiety. Moreover, treatment with ESe prevented the amnesic effect of Sco, as evidenced by increases in the number of entries in the top zone of the tank (Figure 6A, *p* < 0.0001 for 1 mg/L) as compared to the Sco-alone treated group, whereas a significant increase in the time spent in the top zone of the tank was noticed for the 1 mg/L (Figure 6B, *p* < 0.01).

**Figure 7:**
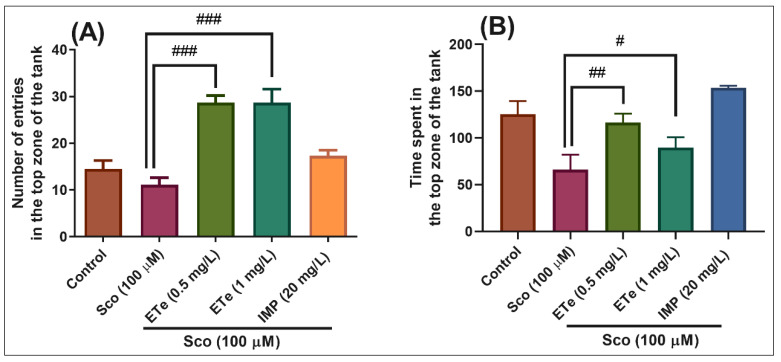
The *Equisetum telmateia* ethanolic extract (ETe, 0.5 and 1 mg/L) reduced the anxiety-like response in the scopolamine (Sco, 100 μM)-induced zebrafish in the novel tank diving (NTT) test. (**A**) represent the number of entries in the top zone of the tank in different groups; (**B**) represent the time spent in the top zone of the tank (s) in different groups. For Tukey’s posthoc analysis: Sco vs. ETe (0.5 mg/L): ### *p* < 0.0001; Sco vs. ETe (1 mg/L): ### *p* < 0.0001 (**A**); Sco vs. ETe (0.5 mg/L): ## *p* < 0.001; Sco vs. ETe (1 mg/L): # *p* < 0.01 (**B**).

In the NTT test, one-way ANOVA demonstrated a significant effect of the treatment on the number of entries in the top zone of the tank in different groups (F(4,45) = 18.21, *p* < 0.0001) (Figure 7A), and the time spent in the top zone of the tank (F(4,45) = 8.35, *p* < 0.0001) (Figure 7B). Exposure to Sco resulted in a significant decrease in the time spent in the top zone of the tank as compared to the control group (Figure 7B, *p* < 0.001), suggesting high levels of anxiety. Moreover, treatment with ETe prevented the amnesic effect of Sco, as evidenced by increases in the number of entries in the top zone of the tank (Figure 7A, *p* < 0.0001 for 0.5 mg/L and *p* < 0.0001 for 1 mg/L) as compared to the Sco-alone treated group, whereas a significant increase in the time spent in the top zone of the tank was noticed (Figure 7B, *p* < 0.001 for 0.5 mg/L and *p* < 0.01 for 1 mg/L).

Our results are in line with other data reported by different authors about the anxiolytic effects of *Equisetum* extracts. Vieira et al. [29] demonstrated that the extract of *E. arvense* disrupted the behavioral states (fear- and anxiety-like disorders) noticed in zebrafish. Singh et al. [30] reported that ethanolic extract of *E. arvense* exhibited anxiolytic effects in mice, due to the flavonoid content. Sarris et al. [31] nicely described in a review of preclinical studies the anxiolytic profile of the *E. arvense,* describing a mechanism that primarily involved gamma-aminobutyric acid (GABA), either via direct receptor binding or ionic channel or cell membrane modulation. Additionally, it has been stated that (-)-epicatechin mitigated hippocampus oxidative stress, anxiety-like behavior, and systemic inflammation in aged mice [32]. Park et al. [33] provided evidence that (-)-epigallocatechin-3-O-gallate reversed caffeine-induced anxiogenic-like effects in an animal model. Vignes et al. [34] also reported that (-)-epigallocatechin-gallate induced anxiolytic activity in mice which could result from an interaction with GABA(A) receptors. Gadotti et al. [35] demonstrated the anxiolytic effects of the flavonoid luteolin in a mouse model of acute colitis. Luteolin compound exerted a significant antidepressant effect at a low dose and could be considered as a novel therapeutic strategy in depression as reported by Crupi et al. [36]. Furthermore, luteolin has antidepressant-like effects, partly due to the suppression of endoplasmic reticulum stress [37]. Zhang et al. [38] reported that quercetin exerted the beneficial or detrimental effects on the shoaling and anxiety behaviors in zebrafish depending on the treatment concentrations, and the underlying mechanisms are potentially associated with neuroinflammation and neuron apoptosis. Quercetin inhibited anxiety-like symptoms and neuroinflammation induced by lipopolysaccharide in rats as stated by Lee et al. [39]. Quercetin mitigated anxiety-like behavior and normalized hypothalamus-pituitary-adrenal axis function in a mouse model of mild traumatic brain injury as reported by Kosari-Nasab et al. [40]. Furthermore, quercetin protected against stress-induced anxiety- and depression-like behavior and improved memory in male mice as shown by Samad et al. [41]. Salgueiro et al. [42] suggested that apigenin possessed anxioselective effects, acting on central benzodiazepine receptors as an anxiolytic agent. Ahmad et al. [43] reported the ability of kaempferol to inhibit the anxiety state in rats. Furthermore, it was demonstrated that chlorogenic acid had anxiolytic effect coupled with antioxidant activity. Caffeic acid also produced antidepressive- and/or anxiolytic-like effects through indirect modulation of the alpha 1A-adrenoceptor system in mice as reported by Takeda et al. [44]. Finally, ferulic acid through mitigation of NMDA receptor pathway exerted an anxiolytic-like effect in mouse model of maternal separation stress [45]. Upon these reports, our *Equisetum* extracts also exhibited an anxiolytic profile in the Sco zebrafish model that could be attributed to their flavonoid and polyphenolcarboxylic acids content.

#### 2.4.2. Y-Maze Test

The test is based on the tendency of zebrafish to explore a new environment and assess whether the exploratory behavior is modulated by past experiences [46]. For each type of the *Equisetum* ethanolic extract (EPe, ESe, ETe), individual tests were performed at both 0.5 mg/L and 1 mg/L dose.

**Figure 8:**
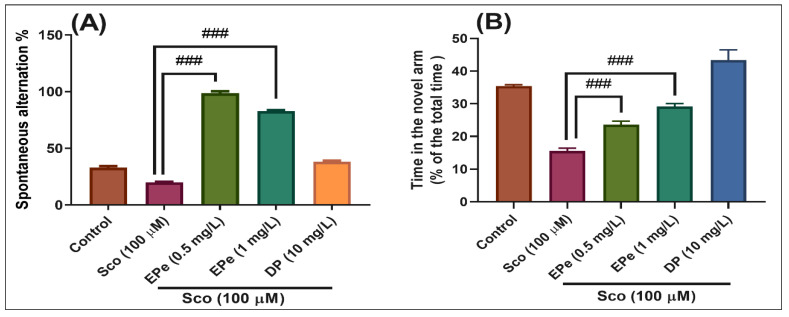
Behavioral response to the *Equisetum pratense* ethanolic extract (EPe, 0.5 and 1 mg/L) treatment in the scopolamine (Sco, 100 μM)-induced zebrafish in Y-maze test. (**A**) represent the spontaneous alternation percentage in different groups; (**B**) represent the time in the novel arm (% of the total time) in different groups. For Tukey’s posthoc analysis: Sco vs. EPe (0.5 mg/L): ### *p* < 0.0001; Sco vs. EPe (1 mg/L): ### *p* < 0.0001 (**A**); Sco vs. EPe (0.5 mg/L): ### *p* < 0.0001; Sco vs. EPe (1 mg/L): ### *p* < 0.0001 (**B**).

In the Y-maze test, the results of the one-way ANOVA revealed a significant effect of the treatment on the spontaneous alternation percentage (F(4,45) = 32.40, *p* < 0.0001) (Figure 8A) and time in the novel arm (% of the total time) F(4,45) = 45.50, *p* < 0.0001) (Figure 8B). Administration of Sco altered the spatial memory formation, as evidenced by a decrease in the percentage of the spontaneous alternation (*p* < 0.0001) (Figure 8A), whereas the response to novelty evaluated through the time in the novel arm, was impaired (*p* < 0.0001) (Figure 8B). By contrast, treatment with EPe, acted against Sco-induced amnesia as noticed by increasing the spatial memory formation (significant increase of the spontaneous alternation percentage, *p* < 0.0001 for 0.5 mg/L and *p* < 0.0001 for 1 mg/L) (Figure 8A) and the time in the novel arm (*p* < 0.0001 for 0.5 mg/L and *p* < 0.0001 for 1 mg/L) (Figure 8B) as compared to Sco-alone treated groups. Donepezil (DP, 10 mg/L), a cholinesterase inhibitor, was used as a reference drug.

**Figure 9:**
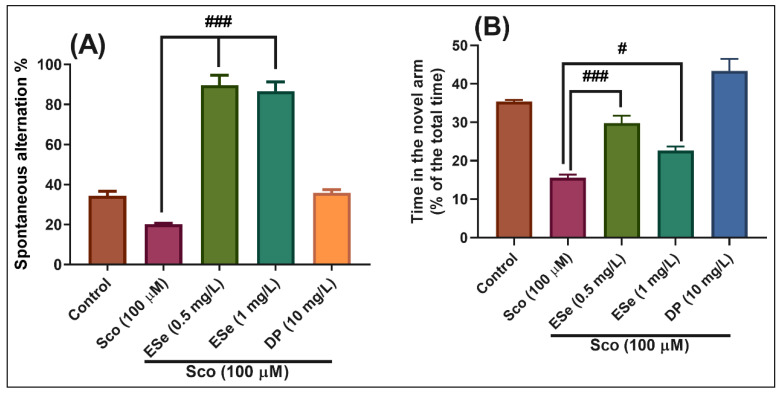
Behavioral response to the *Equisetum sylvaticum* ethanolic extract (ESe, 0.5 and 1 mg/L) treatment in the scopolamine (Sco, 100 μM)-induced zebrafish in Y-maze test. (**A**) represent the spontaneous alternation percentage in different groups; (**B**) represent the time in the novel arm (% of the total time) in different groups. For Tukey’s posthoc analysis: Sco vs. ESe (0.5 mg/L): ### *p* < 0.0001; Sco vs. ESe (1 mg/L): ### *p* < 0.0001 (**A**); Sco vs. ESe (0.5 mg/L): ### *p* < 0.0001; Sco vs. ESe (1 mg/L): # *p* < 0.01 (**B**).

In the Y-maze test, the results of the one-way ANOVA revealed a significant effect of the treatment on the spontaneous alternation percentage (F(4,45) = 97.53, *p* < 0.0001) (Figure 9A) and time in the novel arm (% of the total time) F(4,45) = 38.77, *p* < 0.0001) (Figure 9B). Administration of Sco altered the spatial memory formation, as evidenced by a decrease in the percentage of the spontaneous alternation (*p* < 0.01) (Figure 9A), whereas the response to novelty evaluated through the time in the novel arm, was impaired (*p* < 0.0001) (Figure 9B). By contrast, treatment with ESe, attenuated the Sco-induced amnesia as evidenced by increasing the spatial memory formation (significant increase of the spontaneous alternation percentage, *p* < 0.0001 for 0.5 mg/L and *p* < 0.0001 for 1 mg/L) (Figure 9A) and the time in the novel arm (*p* < 0.0001 for 0.5 mg/L and *p* < 0.01 for 1 mg/L) (Figure 9B) as compared to Sco-alone treated groups.

**Figure 10:**
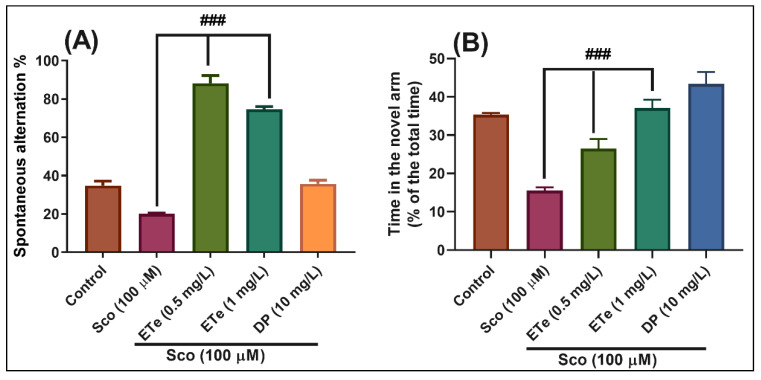
Behavioral response to the *Equisetum telmateia* ethanolic extract (ETe, 0.5 and 1 mg/L) treatment in the scopolamine (Sco, 100 μM)-induced zebrafish in Y-maze test. (**A**) represent the spontaneous alternation percentage in different groups; (**B**) represent the time in the novel arm (% of the total time) in different groups. For Tukey’s posthoc analysis: Sco vs. ETe (0.5 mg/L): ### *p* < 0.0001; Sco vs. ETe (1 mg/L): ### *p* < 0.0001 (**A**); Sco vs. ETe (0.5 mg/L): ### *p* < 0.0001; Sco vs. ETe (1 mg/L): ### *p* < 0.0001 (**B**).

In the Y-maze test, the results of the one-way ANOVA revealed a significant effect of the treatment on the spontaneous alternation percentage (F(4,45) = 151.50, *p* < 0.0001) (Figure 10A) and time in the novel arm (% of the total time) F(4,45) = 27.01, *p* < 0.0001) (Figure 10B). Administration of Sco altered the spatial memory formation, as evidenced by a decrease in the percentage of the spontaneous alternation (*p* < 0.0001) (Figure 10A), whereas the response to novelty evaluated through the time in the novel arm, was impaired (*p* < 0.0001) (Figure 10B). By contrast, treatment with ETe attenuated the Sco-induced amnesia as evidenced by increasing the spatial memory formation (significant increase of the spontaneous alternation percentage, *p* < 0.0001 for 0.5 mg/L and *p* < 0.0001 for 1 mg/L) (Figure 10A) and the time in the novel arm (*p* < 0.001 for 0.5 mg/L and *p* < 0.0001 for 1 mg/L) (Figure 10B) as compared to Sco-alone treated groups.

Supporting data from the literature indicated cognitive enhancement effects of *Equisetum* extracts. Dos Santos Jr et al. [47] demonstrated that cognitive enhancement in aged rats after chronic administration of *E. arvense* L. may be attributed, at least in part, to its antioxidant action. Itoh et al. [48] demonstrated that epicatechin increased the persistence of long-term memory formed by conditioned taste aversion in *Lymnaea stagnalis*. (-)-Epicatechin mitigated high fat diet-induced neuroinflammation and altered behavior in mice as reported by Kang et al. [49]. Diaz et al. [50] demonstrated that epicatechin reduced spatial memory deficit caused by amyloid-β25-35 toxicity modifying the heat shock proteins in the ca1 region in the hippocampus of rats. Tan et al. [51] reported that luteolin treatment effectively alleviated brain edema and ameliorated neurobehavioral dysfunction and memory loss in vivo. Richetti et al. [52] reported that quercetin and rutin prevented scopolamine-induced memory impairment in zebrafish. Sang et al. [53] reported that novel apigenin-rivastigmine hybrids could improve scopolamine-induced memory impairment in zebrafish. Hussein et al. [54] evidenced the neuroprotective role of kaempferol against chlorpyrifos-induced oxidative stress and memory deficits in rats via GSK3β-Nrf2 signaling pathway. Furthermore, chlorogenic acid improved the spatial memory of rats and prevented the CA1 pyramidal cell death after bilateral common carotid occlusion by increasing Bcl2, SOD2, and CD31 expressions and decreasing ET-1 expression [55]. Deshmukh et al. [56] reported that caffeic acid attenuated oxidative stress, learning and memory deficit in intra-cerebroventricular streptozotocin induced experimental dementia in rats. Finally, ferulic acid exerted Nrf2-dependent protection against prenatal lead exposure-induced cognitive impairment in offspring mice as reported by Yu et al. [57]. Our *Equisetum* extracts also exhibited a cognitive-enhancing profile in the Sco zebrafish model that could be attributed to their flavonoid and polyphenolcarboxylic acids content.

When correlating the chemical composition with the biological tests performed in vitro (antioxidant action, enzymatic inhibitors, radical scavenger), there is a direct reciprocity relationship between the increased concentration of flavonoids, the doses administered, and the anxiolytic and antidepressant intensity in the fish. Moreover, considering the high level of silicon (water-soluble silicon derivatives) of the species *E. telmateia*, it was observed that the efficacy of the extracts was below the level of the other two samples. When evaluating the effect on short-term memory, significant differences were observed that reconfirm the great effectiveness of the *E. pratense* and *E. telmateia* extracts compared to *E. sylvaticum*.

## 3. Materials and Methods

### 3.1. Plant Materials

Larger quantities (2 kg) of *E. pratense* and *E. sylvaticum* were harvested from the North-Eastern region of Romania in June–July 2017. *E. telmateia* (2 kg) was collected in July 2018 also from the North-Eastern region of Romania. The botanical identity was checked and attested in the plant biology laboratory by Acad. Prof. Toma Constantin PhD from the Biology Faculty, “Al. I. Cuza” University Iasi Romania. After identification, the raw material was dried to a constant mass for 21 days in a single layer in a dark room at a controlled temperature of 23 °C. To obtain the extracts, the aerial parts of the *Equisetum* species were used. Methanolic extracts were obtained from 10 g of homogenized 2 kg of dry plant material with methanol 70% using the water bath and the refrigerant for 60 min. The obtained methanol extracts were evaporated using the rotary evaporator Buchi R10 (Flavil, Switzerland) [58,59]. Ethanolic extracts were obtained from 10 g of homogenized 2 kg of dry plant material with ethanol 70% using an ultrasonic bath (Bandelin Sonorex, Type RK 31, Berlin, Germany) at 30 °C for 30 min. The mixture was filtered and then the ethanolic extracts were evaporated. All dry extracts were brought to a constant weight so that there was no residual water. The dried extracts were then brought into a vial and maintained for analysis in a dry, cool, dark place [5,60]. The extracts were homogenous, fine glassy fragments were present for the ethanolic samples. The homogeneity of the analyzed extracts was ensured by fine powdering each sample in a glass mortar.

### 3.2. UHPLC Analysis of Phenolic Acids and Flavonoids

The ultra-high-performance liquid chromatography (UHPLC) analysis of the investigated extracts aimed to highlight the most important active principles in the class of polyphenols. Thus, the analysis used the system Thermo Fischer UltiMate 3000 (Pro Analysis Systems, Bucharest, Romania) coupled with a quaternary pump (LPG-3400 SD) with degasser built-in four-channel, autosampler (allows analysis of consecutive 120 samples) and the detector type MULTIDIODE (DAD UltiMate 3000RS). This equipment is adapted to reach a pressure of 62 MPa/9000 psi/620 bar, which allows a fast running of the analysis method in a relatively short time (max. 25 min). Luna PFP (150 × 4, 6 × 4) (Phenomenex, CA, USA) was the column attached to this system. For the detection, the corresponding data were recorded at 5 different wavelengths, using the two lamps, and the detection range was between 190 nm and 800 nm (UV-VIS) to highlight the different types of polyphenols present in extracts included in the study [17,61]. The used standards (purity ≥ 97% HPLC grade, Sigma-Aldrich, Munich, Germany) were: chlorogenic acid, caffeic acid, ferulic acid, rosmarinic acid, p-coumaric acid, epicatechin, quercetin-3-glucoside, apigenin-7-glucoside, apigenin, quercetin, luteolin, luteolin-7-glucoside, kaempferol, and rutoside (5–10 µg/mL in methanol). The solvent system consisted of a mixture of acetonitrile with 0.1% phosphoric acid and 0.1% phosphoric acid. Samples were injected in amounts of 5 μL along with the standards. The data were integrated with the Thermo Scientific ™ Dionex ™ Chromeleon ™ v. 7.2.12 program, with dynamic algorithms for identifying the tracked peaks, and then the results were compared with the databases in the specialized literature. The identity of each compound was confirmed both using the reference spectra and the retention time. Moreover, calibration curves (r^2^ = 0.9989) were obtained for chlorogenic acid, luteolin-7-glucoside, and quercetin-3-glucoside. Triplicate analysis was performed for each sample. The limit of detection (LOD) was calculated at 280 ng/mL and the limit of quantification (LOQ) was 145 ng/mL.

### 3.3. Antioxidant Capacity Assay

The following tests were used to study antioxidant activity: chelation of ferrous ion, lipoxygenase inhibition, the scavenger capacity of the hydroxyl radical, and the superoxide radical anion. The chelating capacity of the ferrous ion was determined using ferrozine which forms a pink complex with maximum absorbance at 562 nm [62]. Determination of the lipoxygenase inhibition capacity could be measured because the active compounds present in the extracts block 15-lipoxygenase by blocking linoleic acid oxidation and reducing absorbance at 234 nm [63]. Determination of the scavenger action of the hydroxyl radical is based on the method that hydroxyl radical, formed in the reaction between ferrous ion and hydrogen peroxide, will hydroxylate salicylic acid to form a pink-violet compound with maximum absorbance at 562 nm [64]. The scavenger capacity of the superoxide anion was determined because the superoxide radical generated by the reduced nicotinamide adenine nucleotide-phenazine methosulfate system reduces nitro blue tetrazole to a violet-blue formazan with a maximum absorbance at 560 nm [65]. All quantifications represent the mean values ± SD (standard deviation) for 5 consecutive determinations, calculated in Excel, *p* = 0.0016, which indicates a statistic significance.

### 3.4. Animals and Group Division

100 adult male and female, wild-type short-fin zebrafish (*Danio rerio*), 3–5 months old were obtained from Pet Product S.R.L., Bucharest, Romania. Zebrafish, approximately 1:1 female to male were maintained in three tanks of 70 L each, an automatic filtration system, and controlled aeration. Fish were fed two times daily with NovoMalawi (JBL, Neuhofen, Germany). The animal room was illuminated on a 14/10 h light/dark cycle. Room temperature was maintained at 25 °C  ±  1 °C. The water temperature was kept at 27 ± 0.5 °C, pH 7 ± 0.15, dissolved oxygen 6 ± 0.1 mg/L, total ammonia < 0.01 mg/L, total hardness 6 mg/L, and alkalinity of 22 mg/L CaCO_3_. The 10 groups of 10 fish were as follows: control, ethanolic extracts from *E. pratense* Ehrh., *E. sylvaticum* L., and *E. telmateia* Ehrh. (0.5 mg/L and 1 mg/L, respectively), scopolamine (Sco, 100 μM), imipramine (IMP, 20 mg/L), and donepezil (DP, 10 mg/L). Before behavioral tests, fish (except those from the control group) were individually immersed in Sco (100 μM) solution for 30 min to induce amnesia, whereas exposure to *Equisetum* ethanolic extracts (0.5 mg/L and 1 mg/L) was done 1 h before the behavioral tests.

The animals were observed in quarantine at least one week before use in experimental studies. This study was carried out in strict accordance with the recommendations of the Directive 2010/63/EU of the European Parliament and of the Council of 22 September 2010 on the protection of animals for scientific purposes. The protocol was approved by the Faculty of Biology Ethics Committee for animal experimentation (Protocol number 02/30.06.2020). All efforts were made to minimize animal pain and suffering.

### 3.5. Behavioral Assays

Animal behavior was recorded using a Logitech C922 Pro HD Stream webcam (Logitech, Lausanne, Switzerland) and the videos were analyzed using the ANY-maze^®^ behavioral tracking software (Stoelting Co., Wood Dale, IL, USA).

#### 3.5.1. Novel Tank Diving Test (NTT)

In NTT, the zebrafish exhibits robust behavioral responses to novelty-provoked anxiety. The NTT protocol applied in this study was described before by Cachat et al. [66] and Rosemberg et al. [67]. The testing apparatus consisted of a trapezoidal glass tank filled with 1.5 L of home tank water and had the following dimensions: 23.9 cm along the bottom × 28.9 cm at the top × 15.1 cm high with 15.9 cm along the diagonal side, 7.4 cm wide at the top, and 6.1 cm wide at the bottom. The fish were individually placed in the testing tank and their behavior was recorded for 6 min with a webcam placed at 40 cm in the front of the tank. The tank was virtually divided into the top zone and bottom zone, respectively. The following parameters were analyzed: the number of entries in the top zone of the tank and the time spent in the top zone of the tank.

#### 3.5.2. Y-Maze Test

The zebrafish memory and response to novelty were investigated using a protocol of the Y-maze task that was formerly described by Cognato et al. [68] and Zanandrea et al. [69]. The fish were tested in a Y-shaped glass tank, having three arms in size of 25 × 8 × 15 cm (L × l × h) and filled with 3 L of home tank water. Different recognizable geometric shapes, such as triangles, circles, and squares, were placed on the outer walls of each arm. The arms of the maze were set randomly as follows: (i) the start arm (A) from which the fish begins the test, (ii) the other arm (B) that is permanently open, and (iii) the novel arm (C) which is blocked during the training period and open in the testing phase. The center of the Y-maze was not taken into account for the analysis. This task was performed in two stages separated by 1 h between them in order to assess the response to novelty and the spatial recognition memory. During the first stage (training session), the fish was allowed to explore the start and the other arm for 5 min, while the novel arm was kept closed. In the second stage (testing session) the fish was placed in the start arm and was allowed to explore the entire maze for 5 min. The time spent in the novel arm (% of total time), and the spontaneous alternation percentage [70] were the behavioral endpoints examined in this task.

### 3.6. Statistical Analysis

The results were presented as mean ± standard error of the mean (E.S.M.). Behavioral data were analyzed by one-way ANOVA followed by Tukey’s posthoc multiple comparison test, considering treatment as a factor. All analyses were performed by GraphPad software (GraphPad Prism 7.0, La Jolla, CA, USA) and the significance was set at *p* < 0.05.

## 4. Conclusions

The UHPLC analysis of methanolic and ethanolic extracts revealed the presence of the following components: chlorogenic acid, caffeic acid, ferulic acid, neochlorogenic acid, epicatechin, quercetin, quercetin-3-glucoside, luteolin, luteolin-7-glucoside, apigenin-glucoside, and kaempferol. Of all the polyphenolic acids, chlorogenic acid was found in the highest amount in *E. telmateia* species (10.12 mg/g in methanolic extract and 14.38 mg/g in ethanolic extract). Regarding flavonoid derivatives, luteolin-glucoside (11.05 mg/g) stands out in the methanolic extract of *E. sylvaticum* species, while for the ethanolic one, the glycosides of quercetin (41.94 mg/g) stand out. For the species *E. telmateia*, in both extracts, the majority were the glycosides of quercetin and apigenin.

The results obtained in the evaluation of the antioxidant action for the two types of extracts indicate a variable capacity to reduce the oxidative processes. The most effective were the extracts from *E. sylvaticum*, and the weakest were those from *E. pratense*. Thus, it can be highlighted that the flavonoid fraction in these plant products, through glycosylated derivatives, influences the free radical scavenger potential to a greater extent than the polyphenolic acid fraction. The sample, both methanolic and ethanolic of *E. sylvaticum* species, is the richest in flavonoid components, which is why it showed the most intense effects on ferrous ions, hydroxyl radical, and superoxide anion as well as in the inhibition of lipoxygenase. This indicates an increased availability of flavonoids (through the benzopyran-like chemical structure) to bind reactive oxygen species. Correlating the data obtained from the evaluation of the methanolic extract with those obtained from the analysis of the content of polyphenols and flavonoids, the *E. sylvaticum* extract containing the highest number of polyphenols is about three times more active compared to the other two extracts. This difference in activity could be explained by the type of polyphenols present in the samples analyzed, as well as by the large difference in flavonoid content.

The in vivo evaluation of neuroprotective activity was performed by following up the effects produced by the ethanolic extracts of the three species of *Equisetum* above zebrafish in terms of anxiety-like behavior and short-term memory. Novel tank diving test (NTT) analyzed the number of entries to the top zone of the tank and the time spent in the top zone of the tank. The 1 mg/L concentration in *E. pratense* and *E. sylvaticum* had anxiolytic and antidepressant effects higher than the 0.5 mg/L concentration. Regarding the ethanolic extract of *E. telmateia*, the time spent in the top zone of the tank is higher for the concentration of 0.5 mg/L compared to 1 mg/L. Compared to the reference substance, IMP, the number of entries in the top zone of the tank by zebrafish was higher at the concentration of 1 mg/L, while at the concentration of 0.5 mg/L, the number of entries was lower, but comparable with these. The Y-maze test analyzed the percentage of spontaneous alternation and the time spent in the novel arm (% of the total time). The differences between the ethanolic extract concentrations of 0.5 mg/L and 1 mg/L were not significant for the analyzed species. Compared to the reference substance, DP, both the concentrations of 0.5 mg/L and those of 1 mg/L of the three species determined an even twice greater stimulation of short-term memory by increasing the percentage of spontaneous alteration and the response to novelty.

## Figures and Tables

**Table 1:** Flavonoids identified and quantified in the investigated samples.

Compound Expressed in mg/g Plant Product *	Sample (70% Methanolic Extract)			Sample (70% Ethanolic Extract)		
	*E.* *sylvaticum*	*E. pratense*	*E.* *telmateia*	*E.* *sylvaticum*	*E. pratense*	*E.* *telmateia*
epicatechin	0.9010 ± 0.021	0.5925 ± 0.001	1.1967 ± 0.001	0.1586 ± 0.002	0.0263 ± 0.003	0.7079 ± 0.011
quercetin-3-glucoside	1.6995 ± 0.001	8.2442 ± 0.001	23.4765 ± 0.033	41.9429 ± 0.022	3.8440 ± 0.001	13.6175 ± 0.022
luteolin-7-glucoside	11.0456 ± 0.011	1.8562 ± 0.001	5.6447 ± 0.021	3.7656 ± 0.019	0.0378 ± 0.011	1.2254 ± 0.003
apigenin-7-glucoside	2.3249 ± 0.013	2.2947 ± 0.021	27.7463 ± 0.009	7.8806 ± 0.003	1.4626 ± 0.004	21.0042 ± 0.021
luteolin	0.6150 ± 0.011	0.0344 ± 0.003	0.4214 ± 0.009	0.4069 ± 0.011	0.0076 ± 0.021	0.1455 ± 0.011
quercetin	0.0658 ± 0.001	0.0059 ± 0.002	0.4013 ± 0.022	0.0255 ± 0.009	0.0071 ± 0.001	0.1369 ± 0.005
apigenin	0.0716 ± 0.002	0.6260 ± 0.009	0.9571 ± 0.009	0.0591 ± 0.001	0.1359 ± 0.002	0.3758 ± 0.001
kaempferol	1.4997 ± 0.021	0.9781 ± 0.009	1.4417 ± 0.009	0.0216 ± 0.009	0.2022 ± 0.001	0.7609 ± 0.004

* the results represent the average value of triplicate quantification; limit of detection (LOD): 280 ng/mL; limit of quantification (LOQ): 145 ng/mL.

**Table 2:** Polyphenolcarboxylic acids identified in the methanolic and ethanolic extracts of *Equisetum* species.

Compound Expressed in mg/g Plant Product *	Sample (70% Methanolic Extract)			Sample (70% Ethanolic Extract)		
	*E.* *sylvaticum*	*E. pratense*	*E.* *telmateia*	*E.* *sylvaticum*	*E. pratense*	*E.* *telmateia*
neochlorogenic acid	0.2639 ± 0.001	0.0730 ± 0.001	0.6381 ± 0.005	0.4838 ± 0.002	0.1879 ± 0.002	0.3059 ± 0.002
chlorogenic acid	4.3322 ± 0.001	0.7491 ± 0.002	10.1162 ± 0.002	3.6744 ± 0.001	0.7362 ± 0.003	14.3814 ± 0.004
caffeic acid	0.1468 ± 0.002	0.2363 ± 0.002	0.8590 ± 0.003	0.6624 ± 0.002	0.1922 ± 0.001	0.3244 ± 0.002
ferulic acid	0.6414 ± 0.002	0.2295 ± 0.005	1.0245 ± 0.001	5.7962 ± 0.004	0.5559 ± 0.001	1.0439 ± 0.003

* the results represent the average value of average value of triplicate quantification.

## Data Availability

The data presented in this study are available on request from the corresponding author.

## Sample Availability

Samples are available at the Pharmacognosy Department, Facultu of Pharmacy, “Grigore T. Popa” University of Medicine and Pharmacy, Iasi, Romania.
